# A brief survey of tools for genomic regions enrichment analysis

**DOI:** 10.3389/fbinf.2022.968327

**Published:** 2022-10-26

**Authors:** Davide Chicco, Giuseppe Jurman

**Affiliations:** ^1^ Institute of Health Policy Management and Evaluation, University of Toronto, Toronto, ON, Canada; ^2^ Data Science for Health Unit, Fondazione Bruno Kessler, Trento, Italy

**Keywords:** genomic regions enrichment analysis, pathway enrichment analyses, functional annotations, functional enrichment analysis, bioinformatics

## Abstract

Functional enrichment analysis or pathway enrichment analysis (PEA) is a bioinformatics technique which identifies the most over-represented biological pathways in a list of genes compared to those that would be associated with them by chance. These biological functions are found on bioinformatics annotated databases such as The Gene Ontology or KEGG; the more abundant pathways are identified through statistical techniques such as Fisher’s exact test. All PEA tools require a list of genes as input. A few tools, however, read lists of genomic regions as input rather than lists of genes, and first associate these chromosome regions with their corresponding genes. These tools perform a procedure called *genomic regions enrichment analysis*, which can be useful for detecting the biological pathways related to a set of chromosome regions. In this brief survey, we analyze six tools for genomic regions enrichment analysis (BEHST, g:Profiler g:GOSt, GREAT, LOLA, Poly-Enrich, and ReactomePA), outlining and comparing their main features. Our comparison results indicate that the inclusion of data for regulatory elements, such as ChIP-seq, is common among these tools and could therefore improve the enrichment analysis results.

## 1 Introduction

**FIGURE 1:**
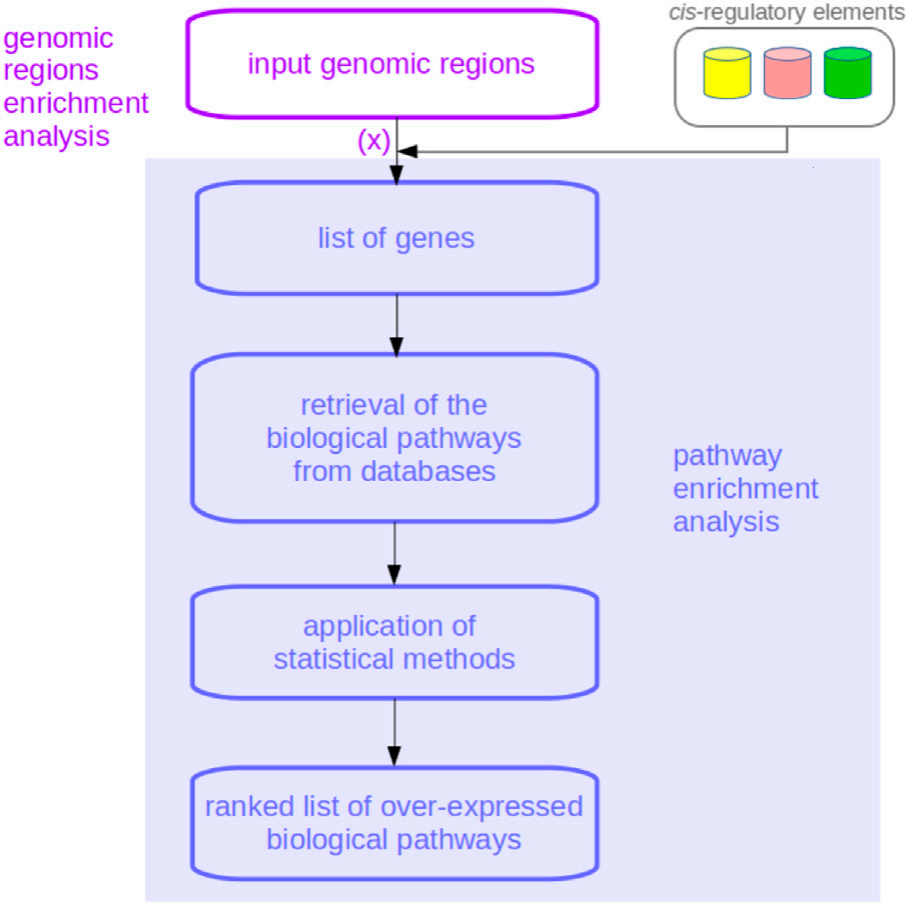
Flowchart of a genomic regions enrichment analysis. The association of the genes corresponding to the query chromosome regions, here indicated with (x), is the phase where BEHST, GREAT, LOLA, and Poly-Enrich involve *cis*-regulatory elements’ data. As is evident, genomic regions enrichment analyses incorporate pathway enrichment analyses.

Pathway enrichment analysis (PEA) methods are a set of bioinformatics techniques and tools which associate biological pathways with gene lists by ranking these pathways based on their over-representation in the list of genes analyzed (Reimand et al., 2019; Mubeen et al., 2022; Chicco and Agapito, 2022). “Gene set enrichment analysis” is another name for PEA, while over-representation analysis (ORA) is an alternative to it (Mubeen et al., 2022; Wijesooriya et al., 2022) which emphasizes the importance of the biological functions which are overrepresented in a gene list with respect to their role in the whole human genome (Chicco and Agapito, 2022). There are many PEA tools (for example, DAVID (Sherman et al., 2022)); they all take genes as input, indicated as symbols or Ensembl ID’s (Spudich et al., 2007). Some other tools, however, read genomic regions and associate genes with these regions before performing PEA (Figure 1). These methods perform genomic regions enrichment analysis, also called chromosome regions enrichment analysis—instead of reading genes as do traditional PEA tools, they read chromosome regions represented as genomic coordinates that are triplets made of a chromosome number, a genomic starting position, and a genomic ending position. The string chr10:134221970-134222202, for example, indicates a genomic region starting at the 134,221,970 position and ending at the 134,222,202 position of chromosome 10 of the genome. Files containing chromosome regions are usually in browser extensible data (BED) format (Quinlan and Hall, 2010; University of California Santa Cruz Genome Browser, 2022). To work properly, genomic region enrichment analysis tools need to specify the genomic coordinates they use as references for genomic regions and the species of interest. GREAT (McLean et al., 2010), for example, accepts data in both the current GRCh38/hg38 genome assembly (National Library of Medicine, 2017b) and in the previous GRCh37/hg19 coordinate reference (National Library of Medicine, 2009) for *Homo sapiens* genomic regions.

Most existing tools for genomic regions enrichment analysis not only associate genes with their corresponding chromosome regions but also consider *cis*-regulatory elements to select more precise and accurate genes, which in turn can lead to more precise and accurate biological pathways as output of functional enrichment analysis.

This short survey lists these tools, describes their main features, and explains how they work and what distinctive traits they have.

We organize the rest of the article as follows. After this Introduction, we list and describe the tools in Section 2 and discuss and compare their features in Section 3.

## 2 Tools

We have found six tools for genomic regions enrichment analysis that currently work: GREAT (McLean et al., 2010), LOLA (Sheffield and Bock, 2016), ReactomePA (Yu and He, 2016), Poly-Enrich (Lee et al., 2020), BEHST (Chicco et al., 2019), and g:Profiler g:GOSt (Raudvere et al., 2019). We found other tools that handle chromosome regions for PEA (Lee et al., 2012; Pageaud et al., 2018) but they seem to no longer work. We list these six tools in Table 1.

**TABLE 1:** List of the genomic regions enrichment analysis tools mentioned in this study. We report each tool’s name, its statistical methods for the *p*-value calculation, its distinctive traits, its web URL, its year, its reference, a field stating whether it is available as web tool, and a field indicating the name of its standalone software package. FDR: false discovery rate. *α*: The LOLA web server is called LOLAweb (Nagraj et al., 2018). g:SCS is the statistical method for adjusted *p*-value calculation employed by g:Profiler g:GOSt by default (g:Profiler g:GOSt, 2022). The article by Raudvere et al. (2019) refers to the latest update of g:Profiler; its original release and previous updates were described in past studies (Reimand et al., 2007, 2011, 2016; Kolberg et al., 2020). The bottom table contains the lists of the supported organisms of the data for each tool.

tool	Statistical methods	Distinctive traits	Web tool
BEHST	g:SCS of g:Profiler g:GOSt	Inclusion of long-range chromatin interactions	Yes
g:Profiler g:GOSt	g:SCS, Bonferroni correction	Fast execution, easy web interface and publication-ready visualization results	Yes
	or Benjamini-Hochberg FDR		
GREAT	Binomial and hypergeometric tests	Inclusion of distal genomic sites of binding events	Yes
LOLA	Fisher’s exact test with FDR correction	Inclusion of transcription factors, histone modifications and DNase hypersensitivity annotations	Yes^*α*^
Poly-Enrich	Likelihood ratio test	Optimized for narrow genomic regions and accounting for the strength of binding sites’ peaks	Yes
ReactomePA	Hyper-geometric tests	Inclusion of molecular reactions and their pathways	No
tool	Web URL	References	softwarepackage
BEHST	https://behst.hoffmanlab.org	Chicco et al. (2019)	behst
g:Profiler g:GOSt	https://biit.cs.ut.ee/gprofiler/gost	Raudvere et al. (2019)	gprofiler2
GREAT	http://great.stanford.edu	McLean et al. (2010)	rGREAT
LOLA	http://lola.computational-epigenetics.org	Sheffield and Bock (2016)	LOLA
Poly-Enrich	http://chip-enrich.med.umich.edu	Lee et al. (2020)	chipenrich
ReactomePA	http://bioconductor.org/packages/ReactomePA	Yu and He (2016)	ReactomePA
tool	Supported organisms		
BEHST	Human		
g:Profiler g:GOSt	Human and 757 other species		
GREAT	Human, mouse		
LOLA	Human, mouse		
Poly-Enrich	Human, mouse, rat, D. melanogaster, D. zebrafish		
ReactomePA	Human, celegans, fly, mouse, rat, yeast, zebrafish		

GREAT (Genomic Regions Enrichment of Annotations Tool) (McLean et al., 2010) is a software program available as a web tool that can be used with any internet browser and as an R package within the Bioconductor platform. GREAT defines a *regulatory domain* for each gene by using its nearest neighbor on either side or a specific genomic distance, annotated to regulatory elements of ChIP-seq data or others. GREAT then overlaps each query chromosome region with these regulatory domains and selects the genes present in them.

LOLA (Locus Overlap Analysis) (Sheffield and Bock, 2016; Nagraj et al., 2018) is a genomic regions enrichment analysis tool available both as a web program (Nagraj et al., 2018) and as an R package within the Bioconductor suite. The most relevant feature of LOLA is its inclusion of regulatory biological elements from the CODEX database (Sánchez-Castillo et al., 2015). These regulatory data include transcription factors that bind sites (as ChIP-seq data), histone modifications (as ChIP-seq data, too), DNase hypersensitivity sites (as DNase-seq data), and transcriptomic information (as RNA-seq data) (Sheffield et al., 2013). LOLA intersects the genomic regions queried by users with the chromosome regions of these regulatory elements, thus facilitating a more precise and accurate selection of genes having a regulatory role.

ReactomePA (Yu and He, 2016) is software package available within the Bioconductor platform which associates the query genomic regions with molecular reactions and pathways from the Reactome database (Gillespie et al., 2022). ReactomePA was built on DOSE (Yu et al., 2015), a disease ontology enrichment analysis package developed and released by the same team.

Poly-Enrich (Lee et al., 2020) is a tool available both online through a browser interface and as a set of software functions within the chipenrich R software package on Bioconductor. The authors built Poly-Enrich on its two predecessors, ChIP-Enrich (Welch et al., 2014) and Broad-Enrich (Cavalcante et al., 2014), which were previously released by the same team. The distinctive trait of Poly-Enrich is that it associates the genes with the query genomic regions by considering the strength of the peaks of the protein binding sites involved as ChIP-seq data. In a nutshell, Poly-Enrich takes into account the association between chromosome regions and genes by observing the strengths of the ChIP-seq peaks—the stronger the peak, the more important are the genes involved around it.

BEHST (Biological Enrichment of Hidden Sequence Targets) (Chicco et al., 2019) is available both as a web tool and as a standalone R package within the Bioconda suite (Grüning et al., 2018). It performs an enhanced association between the query chromosome regions and their corresponding genes by considering long-range interactions in chromatin, included as Hi-C data (Rao et al., 2014). Once BEHST filters in these genes, it sends them as input to g:Profiler g:GOSt, which performs a traditional pathway enrichment analysis through the g:SCS method.

g:Profiler g:GOSt is a popular tool for pathway enrichment analysis which has been mainly used for the association of query gene lists with biomolecular pathways since its first release (Reimand et al., 2007). In the 2019 update of the tool (Raudvere et al., 2019), its developers introduced the possibility of querying not only gene lists but also other data formats, such as chromosome regions, single nucleotide polymorphism (SNP) IDs, and annotation term IDs. g:Profiler g:GOSt therefore now accepts genomic regions, called *chromosomal intervals*, arranged in the BED format according to the GRCh38. p12/hg38 genome reference (National Library of Medicine, 2017a). When reading genomic regions, g:Profiler first retrieves their genes and then performs its traditional functional enrichment analysis. g:Profiler g:GOSt is available both as a web tool and as an R package within CRAN.

## 3 Discussion

The six tools listed and described in this brief survey share some common aspects and have several differences.

ReactomePA and g:Profiler g:GOSt are the only tools among the six in this study which do not make use of data of regulatory elements—BEHST, GREAT, LOLA, and Poly-Enrich all perform a precise selection of the genes involved in the query chromosome regions by using *cis*-regulatory data. ReactomePA and g:Profiler g:GOSt do not apply any filtering step to the input data for the association with the genes—all the genes associated with the input chromosome regions are considered for the functional enrichment analysis. BEHST, LOLA, GREAT, and Poly-Enrich instead use regulatory element data to filter the genes to be employed in the functional enrichment analysis (Table 1).

A user may select which tool to utilize by considering the data involved. GREAT, for example, has a precise definition of a *cis*-regulatory domain that can lead to a more precise selection of the genes that have a regulatory role among the query chromosome regions. Alternatively, LOLA can be selected by a user who would like to have a filtering phase based on multiple regulatory data (ChIP-seq, DNAse-seq, and RNA-seq). Poly-Enrich can be employed by a researcher who wants the filtering to be done not only through regulatory elements’ ChIP-seq data but also considering the strength of their peaks. Users can select BEHST if they are investigating the role of chromatin interactions in a particular study. ReactomePA and g:Profiler g:GOSt, as mentioned above, can be employed if a computational biologist would like to select all the genes related to the query chromosome regions, without any filtering.

Packages’ spread and usage: To better understand the impact of these six tools, we checked their citations in articles, the ratio of citations per month since their publication, and their position in the Bioconductor software package downloads ranking.

These quantitative characteristics show that the articles of g:Profiler g:GOSt, ReactomePA, and LOLA collected the highest amount of citations. In particular, g:Profiler g:GOSt obtained the highest ratio of citations per month since its publication (2,145 citations in total, equal to 53.62 citations per month since the publication of 2019 update study (Raudvere et al., 2019)). However, since g:Profiler g:GOSt can not only be used for genomic regions enrichment analysis but also for traditional pathway enrichment analysis on gene lists, we cannot consider its article the most cited on a genomic regions enrichment analysis tool solely. Regarding the other five tools listed in this survey, which all perform only genomic regions enrichment analysis, it is clear that GREAT has been the most used software program of this category, since it collected 23.65 citations per month and 3,477 citations in total since the publication of its article. ReactomePA was also highly cited (887 to date), its results being the most downloaded software package in Bioconductor among the four available on that platform (128th/2,140).

LOLA’s two articles together count 315 citations on Google Scholar to date (around four citations per month since the publication of the first article of the two), showing that this tool is quite utilized in bioinformatics analyses worldwide. LOLA’s Bioconductor library is 432nd position out of 2,140 in the ranking of the packages’ downloads, while GREAT’s package is ranked 300th in the same standing.

BEHST is the only package among the six listed here that is available on Bioconda and not on Bioconductor or on the Comprehensive R Archive Network (CRAN). Bioconda is a set of bioinformatics software packages available on the Anaconda, Inc. (2020) distributed software platform.

g:Profiler g:GOSt is the only tool among the six available as a software package on CRAN—gprofiler2 (Kolberg et al., 2020). These five Biocondocutor and CRAN software packages were developed in the R programming language (Tippmann, 2015) while BEHST was developed mainly in Python and bash, with a few scripts and a wrapper in R. All six packages were released under free and open-source licenses.

Regarding the enrichment analysis output generated by the six tools under consideration, we noticed that five of them (BEHST, g:Profiler g:GOSt, GREAT, Poly-Enrich, and ReactomePA) produce outcome annotations from similar or the same databases of biomolecular annotations while LOLA produces output terms related to regulatory data only.

g:Profiler g:GOSt generates output annotations from The Gene Ontology, KEGG, Reactome, WikiPathways, Human Protein Atlas, CORUM, TRANSFAC, and miRTarBase. BEHST does too, using g:Profiler g:GOSt as a last step (Table 1). Among the six tools presented in this survey, Poly-Enrich is the tool that finds annotations from the highest number of databases: Biocarta Pathway, KEGG Pathway, Panther Pathway, pFAM, Reactome, Literature Derived, MeSH, MSigDB Derived, Hallmark, Immunologic, Oncogenic, Targets, Comparative Toxicogenomics Database (CTD), Drug Bank, MicroRNA, Transcription Factors, Interaction, Protein Interaction BioGRID, Metabolite, and Cytoband. GREAT in its standard version associates genomic regions with annotations of The Gene Ontology, Ensembl genes, and annotations of Human Phenotype, while ReactomePA—as mentioned above—associates genomic regions only with Reactome pathways. All these five tools (g:Profiler g:GOSt, BEHST, GREAT, Poly-Enrich, and ReactomePA) can be grouped in the same category since they produce output annotations related to The Gene Ontology and other biomolecular databases.

LOLA, on the other hand, is the only tool listed here which produces results related to *cis*-regulatory data, such as from ENCODE transcription factor binding data, DNase hypersensitivity data, Cistrome epigenome data, and CODEX regulatory data, related to the query genomic regions.

Each tool of the six described in this survey produces its output results in its specific way, making it somewhat difficult to compare them computationally. Users could, however, insert the same query genomic region set in all the tools and then study the significance of the shared overrepresented biomolecular annotations produced in the output, such as The Gene Ontology terms.

Applications: Several databases of biologically-relevant chromosome regions now exist for users interested in employing some genomic regions as queries for these tools. One worth mentioning is SlideBase, which provides genomic regions of enhancers and promoters related to specific human tissues and organs (Ienasescu et al., 2016) as GRCh37/hg19 coordinates. For example, chromosome regions of heart enhancers could be downloaded from SlideBase and input into GREAT: the first step of GREAT, as shown in Figure 1, would be associating these input genomic regions with genes near *cis*-regulatory regions. The genes found would then be used in traditional functional enrichment analysis.

**FIGURE 2:**
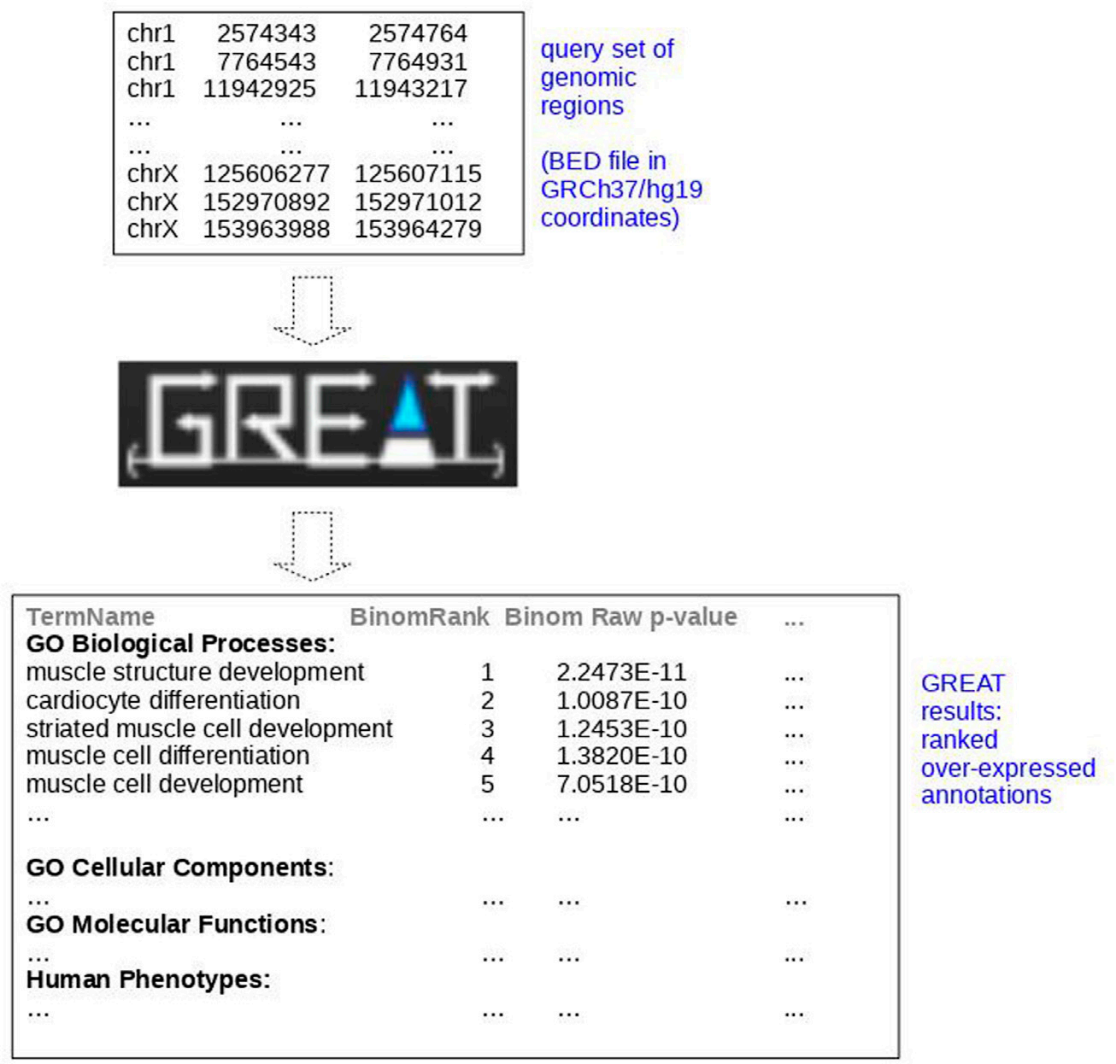
Schematic example of application of GREAT to SlideBase enhancer genomic regions of the heart. Representation of the application of GREAT (McLean et al., 2010) to query genomic regions of the heart downloaded from SlideBase (SlideBase, 2014; Ienasescu et al., 2016) in GRCh37/hg19 coordinates. We obtained these results with GREAT default parameters (basal plus extension, proximal: 5 kilo base pairs upstream, 1 kilo base pairs downstream, plus distal: up to 1000 kilo base pairs). PEA: pathway enrichment analysis, functional enrichment analysis.

We represented a schematic example of an application of GREAT to a set of chromosome regions of heart enhancers available on FANTOM5 (Andersson et al., 2014) downloaded as pre-defined tracks from SlideBase (SlideBase, 2014; Ienasescu et al., 2016) in GRCh37/hg19 coordinates in Figure 2. In the image, we reported some query sets of genomic regions of heart enhancers as input and a few outputs over-expressed as The Gene Ontology (GO) annotations (Ashburner et al., 2000) retrieved by the GREAT web tool. In this example, GREAT found “muscle structure development,” “cardiocyte differentiation”, and “striated muscle cell development” as the most overly expressed GO terms (Figure 2) with their corresponding *p*-values. We reaffirm the importance of using adjusted *p*-values (Chicco and Agapito, 2022) and of setting an adjusted *p*-value threshold to at least 0.005, as suggested by Benjamin et al. (2018). These GO annotations can then be employed to better understand heart biology or for additional computational analyses (Pinoli et al., 2013; Chicco and Masseroli, 2014).

### 3.1 Recap

Below are some key findings arising from the analysis of the six tools listed in this survey.

*Cis*-regulatory elements provide relevant information for genomic regions enrichment analysis. As we already mentioned, four out of the six tools presented in this brief survey involve data of regulatory elements: the authors of these four thus believe that regulatory elements can improve the results of genomic regions enrichment analyses. We will probably see even further involvement of this kind of data in the future.

ChIP-seq data provide relevant information for genomic regions enrichment analysis. Three tools out of the six described in this survey (GREAT, LOLA, and Poly-Enrich) employ chromatin immunoprecipitation DNA sequencing data (ChIP-seq) to include data of the regulatory activities of the genes (Mardis, 2007). This result confirms the importance of ChIP-seq data in computational genomics.

R and Bioconductor are the key programming language and software suite for genomic regions enrichment analysis. As mentioned above, the four tools presented here are available as R software packages on the Bioconductor platform. This aspect proves that R and Bioconductor are key resources for this kind of bioinformatics analysis. Only one of the six software packages mentioned in this study was released on the CRAN official catalogue of R packages (The R Foundation, 2022).

In the future, we envision the development of new tools for genomic regions enrichment analysis, which will likely involve data of regulatory elements such as ChIP-seq, developed both as web tools *via* an internet browser and as R standalone packages within Bioconductor.

## Abbreviations

BED, browser extensible data; BEHST, biological enrichment of hidden sequence targets; ChIP, chromatin immunoprecipitation; CRAN, Comprehensive R Archive Network; FDR, false discovery rate; GREAT, Genomic Regions Enrichment of Annotations Tool; GSEA, gene set enrichment analysis; LOLA, locus overlap analysis; PEA, pathway enrichment analysis; Seq, DNA sequencing; URL, Uniform Resource Locator.
